# Contemporary reflection on the educational levels of high-performance soccer players in Brazil

**DOI:** 10.31744/einstein_journal/2023AO0269

**Published:** 2023-10-10

**Authors:** Paulo Roberto Santos-Silva, Júlia Maria D´Andrea Greve, Renato Luis da Silva, Marcelo Mesquita Spinola

**Affiliations:** 1 Hospital das Clínicas Faculdade de Medicina Universidade de São Paulo São Paulo SP Brazil Laboratório de Estudos do Movimento, Centro Médico de Excelência da FIFA, Instituto de Ortopedia e Traumatologia, Hospital das Clínicas , Faculdade de Medicina , Universidade de São Paulo , São Paulo , SP , Brazil .; 2 R. Soccer Centro de Formação de Atletas São Paulo SP Brazil R. Soccer Centro de Formação de Atletas , São Paulo , SP , Brazil .; 3 Hospital Israelita Albert Einstein São Paulo SP Brazil Hospital Israelita Albert Einstein , São Paulo , SP , Brazil .

**Keywords:** Higher education policy, Soccer, Athletic performance, Athletes, Students, Self-directed learning as topic, Surveys and questionnaires, Educational measurement

## Abstract

**Objective:**

As the soccer culture in Brazil is more popular than schooling, this study reflected on the formal education levels of soccer (football) players through descriptive and quantitative analyses.

**Methods:**

We evaluated 179 national soccer players playing various positions on different teams across six seasons (2012, 2013, 2014, 2015, 2016, and 2022). Data were collected using a questionnaire comprising the following variables: age, position in the pitch, age of professionalization, and education (years of study). The data were distributed according to the pitch position.

**Results:**

The mean age, length of professional career, and professionalization age were 23±6 years, 7±5 years, and 17±2 years, respectively. In terms of education, 121 athletes (67%) completed high school, equivalent to 11 years of study. Only 5.5% completed higher education, with defensive players (goalkeepers and defenders) being the most educated at 37% (66/179). According to 2017 figures from the *Pesquisa Nacional por Amostra de Domicílios Contínua* of the Federal Government of Brazil, the schooling rate is 31.7%, wherein 46.1% of Brazilians aged 25 years or over have completed education. In this study of 179 athletes, 67% had completed high school. Thus, players outrank the Brazilian population in terms of achieving a high school education. There were marked disparities in the relative proportions of goalkeepers (85%), defensive players (68%), midfielders (63%), and forward players (64%) in terms of their education.

**Conclusion:**

Our survey revealed that attending higher education remains a distant reality in Brazil.

## In Brief

Santos-Silva et al. demonstrated that, for Brazilian soccer players, pursuing higher education is still a distant reality. This is because, in relative numbers, only 2.5% of the athletes have completed elementary school, whereas 67% and 5.5% completed high school and higher education, respectively. The mean initial professionalization age is 17.5 years, indicating that soccer players take on a crucial responsibility in a very early stage of life. However, they are still immature in terms of dealing with money,contractual relations with the club, and decision-making.

## Highlights

Only 5.5% of Brazilian soccer players surveyed completed higher education.

Of these 179 athletes, 67% completed high school.

The most educated were goalkeepers and defenders at 37% (66/179).

Physical education for sports is different from educating the mind in terms of brain plasticity.

## Visual Abstract

**Figure:**
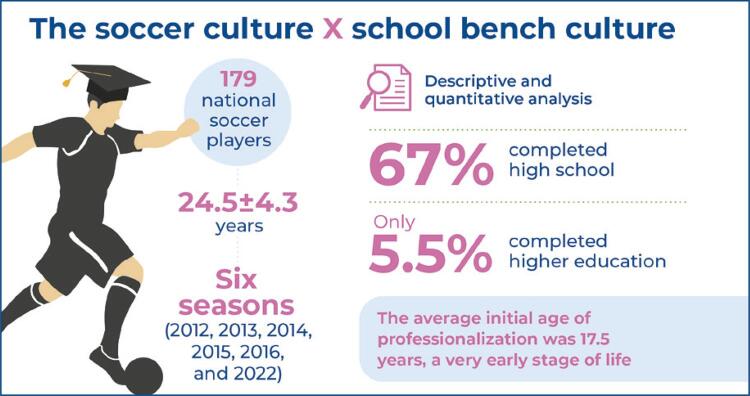

## INTRODUCTION

Soccer players are not renowned for their intelligence and academic prowess. ^( 1 )^ Moreover, research on the academic levels of these athletes is limited. ^( 2 )^ A career in professional soccer is driven by the dreams of athletes and their families. However, young athletes face, and often succumb to, various difficulties and barriers. The illusion of financial success causes great frustration among those who do not achieve stardom. The draw of this sport is in its potential to achieve greater success faster, whereas academic qualification is perceived to be a slower path to achieving financial success compared to playing professional soccer. However, soccer is a team sport that requires rapid decision making and nearly instant thinking to adapt to the unpredictability of the game and can strengthen formal educational knowledge. Soccer (Football) is a game that requires cognition and motor skill to execute, and prior research has found a positive correlation between knowledge and performance. However, the Brazilian education model is not equipped to meet the athletes’ needs. Furthermore, institutions and clubs do not cooperate in implementing a structure because one of the greatest difficulties is releasing athletes from training and games. Few are able to complete their education and break the stereotype of undereducated soccer players.

Thus, the extent to which schooling determines an athlete’s success remains controversial. They must adapt quickly, change strategies, and inhibit their responses. Stratton et al. ^( 3 )^ note that many of these skills are called “game intelligence.” In neuropsychology, Straus et al. ^( 4 )^ scored the collective executive function. However, although these top-down dynamic cognitive processes correlate with each other, they do not correlate with overall IQ. ^( 5 )^ Factors such as quick creativity and problem-solving skills, which require verbal aspects, are highly affected by schooling. ^( 6 )^

Duarte et al. ^( 7 )^ consulted 20 Serie A football teams to determine the level of education of elite national soccer players. Of more than 600 soccer athletes, only 15 attended higher education-just over 2% of the total. The responsibility of balancing training, games, and education, lies partially with the clubs in São Paulo. Law 13,748 of October 8, 2009, assigns to official soccer teams in the State of São Paulo, Brazil, the responsibility for the education of their athletes, as provided in article 1, transcribed below:

Article 1: The Teams’ Official Soccer players of the state must ensure that all players under the age of eighteen are enrolled in an educational institution with whom they have some form of a bond ensuring attendance and school performance. Sole paragraph: – Official teams are associations duly registered and recognized by the São Paulo Soccer (Football) Federation. Article 2: Failure to comply with the obligation set out in the previous article will result in the application of fines and impediments to participation in official tournaments and competitions. 2 - The non-delivery of proof of enrollment and school attendance of players under 18 (eighteen) years of age by official teams to the São Paulo Soccer Federation will denote non-compliance with this law, resulting in the application of penalties. ^( 8 )^

In reality, this situation seems to be related to Brazilian public policies for sports and the education system, where only 16% of formal workers complete higher education. According to data from the National Household Survey (PNAD) released by the Brazilian Institute of Geography and Statistics (IBGE - *Instituto Brasileiro de Geografia e Estatística* ), in the last three months of 2014, three out of every ten people in the Brazilian workforce had not completed elementary school. ^( 8 )^ IBGE data from 2015 show that only 16% of Brazilian workers completed higher education. ^( 8 )^ In 2013, data released by the Organization for Economic Cooperation and Development (OECD) indicated that Brazil had the lowest percentage of higher educated populations and was the third from the bottom in respect of secondary education among the 35 countries surveyed. This harsh reality also affects soccer players. ^( 9 )^ In Brazil, only 57% of adults aged 25–64 years have completed upper secondary education, which is lower than the OECD average of 79%. In addition, only 53% of men have completed high school compared to 60% of women. ^( 10 )^

By contrast, the European Union advocates the creation of education and professional training programs for young, talented people in sports, in parallel with intensive sports training, to prepare them for dual careers. Therefore, the European model is preparatory. All athletes are school-educated, have a career in sports, and attend college. ^( 10 )^

In North America, it is necessary to be a student to participate in competitive sports; in addition, it is required to maintain a certain grade point average in the classroom to be able to play. It is imperative to reconcile the life of a student with that of an athlete in order to participate in competitions. ^( 10 )^ There is a well-adapted program established between the sports coach and the school teacher. Unfortunately, Brazil is not yet ready to offer a compelling model for integrating education and sports. The Ministry of Sports continues to seek inspiration from other countries to implement a more effective model that fits the Brazilian context.

## OBJECTIVE

As the soccer culture in Brazil is more popular than schooling, this study reflected on the formal education levels of soccer (football) players through descriptive and quantitative analyses.

## METHODS

### Study design and participants

The study adopted a cross-sectional, retrospective, exploratory, descriptive design. The Movement Studies Laboratory of the Institute of Orthopedics and Traumatology (Centre of Medical Excellence of FIFA - *Fédération Internationale de Football Association* ) of the Hospital das Clínicas of *the Faculdade de Medicina* of the *Universidade de São Paulo* annually evaluates professional soccer players’ pre-participation in the São Paulo professional soccer championship across its three divisions (A1, A2, and A3). Data were collected through individual interviews post obtaining the approval of the Ethics Committee on Research with Human Beings (case study # CAAE: 7997.75517.7.0000.0065; #2.489.654) of the *Faculdade de Medicina* of the *Universidade de São Paulo* (FMUSP). All participants provided signed informed consent and the research was conducted according to the guidelines set in the Declaration of Helsinki. The sample consisted of 179 male professional soccer athletes distributed by positions played on the pitch: A Group: goalkeepers (20; 11%); B Group: defenders (60; 34%); C Group: midfielders (60; 34%), and D Group: forwards (39; 21%), selected across six seasons (2012, 2013, 2014, 2015, 2016, and 2022) from different professional soccer teams linked to the São Paulo Soccer Federation. The questionnaire used for data collection was constructed by the Movement Studies Laboratory and comprised the following variables: age, position in the pitch, age of professionalization, and education (years of study). Only professional soccer athletes were included in the study.

### Statistical analysis

Qualitative variables are presented as absolute and relative values. Quantitative variables are presented in terms of their central tendency and dispersion values. Normality and homogeneity of variance were obtained using the Kolmogorov-Smirnov and Levene tests, respectively. Parametric tests were used for variables that met these two criteria; otherwise, non-parametric tests were used. ^( 11 )^ The age differences among the groups divided by positions were compared using analysis of variance (ANOVA). Statistical analyses were performed using Sigma Stat (version 3.5, Systat Software, Inc., Point Richmond, CA, USA). Statistical significance was set at p<0.05 (5%).

## RESULTS

The average age of all 179 players interviewed in this study was 24.5±4.3 years; the group-wise distribution of age is shown in table 1 . Of the sample, 67% (121/179) had completed high school, equivalent to 11 years of study. Only 5.5% (10/179) had completed higher education and defensive players were the most educated, with 11% obtaining graduate degrees ( Table 2 ).

**Table 1:** Comparative data on the age of professional soccer players according to the positions played on the pitch

Pitch Position	Mean	Standard deviation	Standard deviation of the mean
Goalkeepers (n=20)	25.6	5.6	1.36
Defenders (n=60)	25.2	4.2	0.57
Midfielders (n=60)	23.8	4.1	0.56
Forwards (n=39)	24.2	4.4	0.79
All (n=179)	24.5	4.3	0.35

ANOVA test: p=0.293.

**Table 2:** Absolute (relative) frequency distribution of education levels among 179 soccer players according to the positions played on the pitch position

Education level	A Group (Goalkeepers) n (%)	B Group (Defenders) n (%)	C Group (Midfielders) n (%)	D Group (Forwards) n (%)
Elementary incomplete	-	-	1 (0.5)	-
Complete Elementary	-	3 (2)	1 (0.5)	-
Incomplete high school	-	1 (0.5)	18 (10)	13 (7)
Complete high school	17 (9)	41 (23)	38 (21)	25 (14)
Incomplete higher education	1 (0.5)	9 (5)	-	1 (0.5)
Complete higher education	2 (1)	6 (3)	2 (1)	-
All athletes/pitch position	20 (100)	60 (100)	60 (100)	39 (100)

Table 2 shows that of 179 athletes, 121 (67%) completed high school. There was a marked disparity noted in the relative proportions for goalkeepers (85%), defensive players (68%), midfielders (63%), and forwards (64%) who had completed high school.

As shown in table 3 , there was no statistically significant difference between the positions in terms of the initial age of becoming a professional soccer athlete.

**Table 3:** Age in years at the beginning of a career as a professional soccer player between positions played on the pitch

Positions	Mean	Standard deviation	Standard deviation of the mean
Goalkeepers, (n=20)	17.1	1.144	0.28
Defenders, (n=60)	17.4	1.295	0.18
Midfielders, (n=60)	17.7	2.068	0.28
Forwards, (n=39)	17.5	1.029	0.18
All (n=179)	17.5	1.552	0.12

ANOVA test: p=0.579.

**Figure 1:**
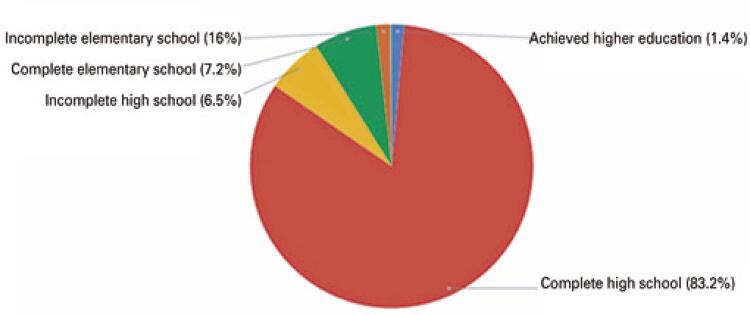
Number of soccer players engaged in 2019 (n=3,329)

Source: https://querobolsa.com.br/revista/1-4-dos-jogadores-de-futebol-contratados-nos-ultimos-meses-cursaram-faculdade

These data provide an overview of the Brazilian players’ levels of education. According to 2017 figures from the National Continuous Household Sample Survey (Pnad C - *Pesquisa Nacional por Amostra de Domicílios Contínua* ) of the Federal Government of Brazil, the schooling rate is 31.7%, whereas 46.1% of Brazilians aged 25 years or older have completed schooling. Further, a larger proportion of athletes completed their high school education as compared to the general Brazilian population ( Figure 1 ).

**Figure 2:**
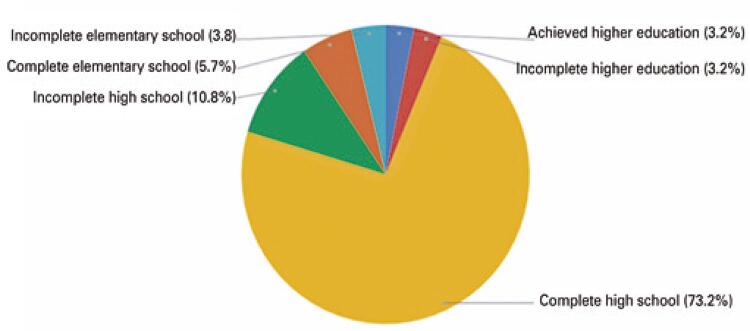
Number of female soccer players engaged in 2019 (n=157)

Source: https://querobolsa.com.br/revista/1-4-dos-jogadores-de-futebol-contratados-nos-ultimos-meses-cursaram-faculdade

Another interesting development is the growth of women’s soccer in Brazil. Moreover, women dominate university education programs in Brazil to an extent. Figure 2 shows that the number of women who moved to the soccer market between December 2018 and March 2019 was smaller than the number of men. This research conducted by the General Register of Employed and Unemployed Persons (CAGED - *Cadastro Geral de Empregados e Desempregados* ), found that of the 157 women offered contracts during this period, ten of them attended college and five reported having a Higher Education diploma.

## DISCUSSION

This study’s primary finding is that most soccer players have completed high school, that is, 11 years of education. Typically, Brazilian soccer athletes have little education and do not complete the 14 years of study indicated by the government. Over the years, the Brazilian educational system has been the target of intense criticism, indicating possible inequalities in access to educational opportunities. Furthermore, improving the levels of education and equality among the Brazilian population has been an insurmountable challenge. Currently, 96.5% of Brazilian children aged 7–14 years are enrolled in schools, suggesting some progress. ^( 12 )^ However, indicators show that almost all public school students across all levels perform below the standard prescribed by their respective grades, and many remain functionally illiterate. ^( 12 )^ This information is validated by the poor performance of students in the National System of Evaluation of Basic Education (SAEB - *Sistema Nacional de Avaliação da Educação Básica* ) in Brazil and the Program for International Student Assessment (PISA) examination. Program for International Student Assessment was created and developed in 1997 by the German researcher, Schleicher, who started a new OECD approach to education. ^( 12 )^

This difficulty is also encountered in soccer. In all positions, the percentage corresponding to each advancing level of education was very small, indicating an interruption in school activities after reaching high school ( Table 3 ). In a similar vein, the literature identifies boxing as the sport with the lowest level of education. ^( 13 )^ In the boxing field, more than 40% end their education in elementary school, and none of the athletes surveyed had enrolled in a higher education course. Football players prefer courses in physical education, administration, and law. ^( 14 )^ Our results also show that goalkeepers are more likely to attend university. We found defenders and goalkeepers had the greatest increase in university education, although in small percentages ( Table 2 ).

Indisputably, technical skill, creativity, and innate talent are matters of great relevance in soccer and other team sports. However, it remains unclear whether athletes with a low general educational level have poor cognitive sports performance. This aspect of the game is related to the response selection processes and, thus, the athlete uses their cognitive ability to read the game. It is important to emphasize that the knowledge athletes use in team sports is specific and not provided in general school education. ^( 15 , 16 )^ According to Sternberg, ^( 17 )^ three types of mental skills constitute intelligence: analytical, creative, and practical. Therefore, school intelligence does not always correlate with the motor intelligence required for sports. Nevertheless, we cannot neglect that the tactical part of the game is linked to an athlete’s analytical repertoire. Therefore, whether in learning the rules of the game or in the skills necessary for its performance, cognitive aspects involving memory and attention are fundamental. ^( 18 )^ Soccer is considered a complex sport requiring rapid action and reaction, due to unpredictability in space and time, with frequent technical variations, random actions, and multiple choices. ^( 19 )^ These conditions lead players to frequent choices of recurrent behaviors of cognitive and perceptual abilities that seem to be determinants of their performance in the game. ^( 20 )^ As a result, mastering cognitive skills can be a deciding factor for athletes to improve their performance by reducing decision and reaction times, enabling them to anticipate opponent plays more easily. ^( 19 )^ According to Ali, ^( 15 )^ the ability to read the game with consequent appropriate decision making is considered a determining factor for a player’s success. When one considers and knows how to interpret information, such as their position and that of their teammates and opponents, location and trajectory of the ball, distance to the goal or field limit, instructions from your coach, and field alertness, among others, they can improve their reading and assimilation of game signals within fractions of seconds and take more accurate decisions in the face of objectives and challenges. ^( 21 , 22 )^ As can be seen in this study, players are too young to manifest maturity in terms of cognitive talent ( Table 2 ). The sporting quality of an athlete is structured into two performance components: cognitive and motor. ^( 21 , 22 )^ According to Thomas et al., ^( 23 )^ soccer, as a collective game, involves different cognitive processes such as perception, attention, anticipation, memory, thinking, and intelligence, all related to each other and supported by declarative and procedural knowledge structures. The pursuit of these requirements to be an above-average soccer athlete is not an easy task for such young players. Given the difficulties in observation of training and games, this may be one of the reasons that justify the figure of talent scouts in clubs. However, this method is extremely subjective, and, as much as the talent scout has knowledge of the pitch, the choice is often based on their personal intuition than on objective criteria. ^( 24 )^ However, training the body for playing a sport is different from educating the mind with regard to brain plasticity.

The coordinator of the coaching courses of the Brazilian Soccer Confederation (CBF - *Confederação Brasileira de Futebol* ) believes that the level of education of professional soccer players is capable of influencing their performance on the pitch, and thereby the team’s performance:

“The ability to interpret the coach’s speech, training issues, sport science, and the scientific context behind training all have to do with the player’s educational level, and cognitive and social development. There are other ways of completing high school and university education that are suitable for athlete training”.

In another statement by Marques, the CBF director, he said:

“There are studies that indicate that, by having an education, the athlete is able to make a more complete plan for t post-sports career, providing them with more options. There are players who stop playing and have no perspective, and the level of education is directly linked to this issue”.

More recently, data from the CAGED survey conducted in Brazil in 2019, with 3,329 soccer players hired, showed that just over 1% (47 athletes) had access to higher education. ^( 25 )^ When compared with the results of this study, we noticed a worsening trend, since the relative participation in higher education was 6% in 2016, and dropped to 1% in 2019 ( Table 2 ). Regarding sporting success, most will not be sufficiently rewarded to ensure an income or financial security beyond their retirement from athletics. For these athletes, a second career after retirement, and a corresponding better education, may be a necessity. However, in modern sports, this presents many challenges. ^( 26 )^ Many athletes wait until their retirement to start thinking about what they should do next. High-performance sport has the cruel side of inhibiting the athlete’s ability to study and compete during his professional life. ^( 27 )^ Education is certainly a great plan that should go along with athletic activities; however, sport is cruel and demands the best from the athlete in every domain. A mind not nourished by education quickly succumbs to the desire for financial success, particularly when the athlete comes from a lower socio-economic level. ^( 26 )^

This study suggests that soccer institutions in Brazil prioritize playing soccer, forgetting the rights guaranteed to athletes as citizens. The priority, on the contrary, should be education, which is essential for personal development and growth. ^( 28 )^ It seems that there is a disconnect between the schools and soccer clubs, with neither organization taking responsibility to oversee the school and sports performance of these young athletes or monitor and measure the evolution and results of the two institutions. Considering the difficulties that the sport imposes on athletes, special allowances regarding the requirements in tasks, evaluations, frequency, and even curriculum and its development (over time), should be provided as incentives in an attitude of inclusion and respect for differences justified by the sports profession. ^( 29 - 31 )^ We propose that promoting the combination of study and sport should be a task for the institution’s decision-makers within an ecological framework that integrates quantitative and qualitative methodologies.

## CONCLUSION

In conclusion, our initial survey revealed that attending higher education remains a distant reality for professional football players because, in relative numbers, very few of the athletes completed elementary school, whereas a few more completed high school and a small number completed higher education. The average age of initial professionalization was 17.5 years. This indicates that these athletes, who are still not completely mature, must assume great responsibility in terms of dealing with money, social and contractual relationships with the club, and decision making. A career in professional sports, and the fame and fortune that accompanies it, is the dream of many young athletes. In reality, few make it to the big league, wherein, despite the rewards and recognition, financial success is still not guaranteed. Therefore, financial literacy is key for young to help them make the right investment decisions early. Training athletes for additional professional qualifications will provide them with security when it comes to ending a sports career and entering the market.
